# Increased pulsatility index of the basilar artery is a risk factor for neurological deterioration after stroke: a case control study

**DOI:** 10.1186/s40885-022-00210-9

**Published:** 2022-08-15

**Authors:** Il-Han Yoo, Jeong-Min Kim, Su-Hyun Han, Jaiyoung Ryu, Keun-Hwa Jung, Kwang-Yeol Park

**Affiliations:** 1 Department of Neurology, Nowon Eulji Medical Center, Eulji University School of Medicine, Seoul, Republic of Korea; 2 Department of Neurology, Seoul National University Hospital, Seoul National University College of Medicine, Seoul, Republic of Korea; 3 Department of Neurology, Chung-Ang University Hospital, Chung-Ang University College of Medicine, Seoul, Republic of Korea; 4 Department of Mechanical Engineering, Chung-Ang University, Seoul, Republic of Korea

**Keywords:** Basilar artery, Stroke, Transcranial Doppler sonography, Neurological deterioration

## Abstract

**Background:**

Higher pulsatility of the middle cerebral artery (MCA) is known to be associated with stroke progression. We investigated whether pulsatility index (PI) of the basilar artery (BA) can predict neurological deterioration (ND) after acute cerebral infarction.

**Methods:**

A total of 708 consecutive patients with acute ischemic stroke who had undergone transcranial Doppler (TCD) ultrasonography were included. ND was defined as an increase in the National Institutes of Health Stroke Scale scores by two or more points after admission. The patients were categorized into quartiles according to BA PI. Multivariable logistic regression analysis was performed to examine whether BA PI is independently associated with ND.

**Results:**

BA PI was well correlated with the right (*n =* 474, r^2^ = 0.573, *P <* 0.001) by Pearson correlation analysis although MCA PI could not be measured from right MCA (*n =* 234, 33.05%) and left MCA (*n =* 252, 35.59%) by TCD owing to insufficient temporal bone window. Multivariable logistic regression analysis including age, sex, cerebral atherosclerosis burden, National Institutes of Health Stroke Scale at admission, and the proportion of patients with current smoking status, hypertension, diabetes mellitus, atrial fibrillation revealed that the higher BA PI (odds ratio, 3.28; confidence interval, 1.07–10.17; *P =* 0.038) was independently associated with ND.

**Conclusions:**

BA PI, which would be identified regardless of temporal window, could predict ND among acute stroke patients.

**Supplementary Information:**

The online version contains supplementary material available at 10.1186/s40885-022-00210-9.

## Background

Neurological deterioration (ND) occurs in 10 to 58% adult stroke patients and results in poor prognosis and mortality [1–5]. Several factors are known to be associated with ND, such as old age, diabetes mellitus (DM), hypertension (HTN), smoking habit, coronary heart disease, the size of low-density lesions as observed on initial computed tomography (CT), change in the flow velocity of middle cerebral artery (MCA), impaired cerebral hemodynamic reserve, blood glucose level, proinflammatory cytokine level, and blood pressure (BP) [5–10]. From a mechanistic perspective, failed intracranial collateral blood flow or elevated intracranial pressure may lead to decreased cerebral perfusion, thereby causing ND [2].

Because the stiffness of large arteries is linked with various cerebral small vessel disease phenotypes including cerebral microbleeds, white matter hyperintensities, and lacunar cerebral infarction, it may be plausible that increased cerebral arterial stiffness is associated with ND after stroke [11–17]. The pulsatility index (PI) of intracranial cerebral arteries, as measured by transcranial Doppler (TCD) ultrasonography, is known to reflect the resistance of downstream arteries and compliance of large cerebral arteries [17–20]. Recent study reported that elevated MCA PI is independently associated with ND among lacunar stroke patients [17]. However, in 18% of patients with acute ischemic stroke or transient ischemic attack (TIA), MCA PI is unobtainable due to poor acoustical temporal bone window; It is known that a poor acoustical temporal bone window is more common in not only elderly patients, but also female patients and those with thick skull [21].

The basilar artery (BA) PI can be measured through the transforaminal approach, which the transducer is placed just below the occipital protuberance and directed towards the nasal bridge. However, the clinical significance of BA PI among stroke patients has not been appreciated yet. We investigated whether BA PI can predict ND after acute stroke.

## Methods

### Patients and evaluation

From January 2014 to December 2015, consecutive patients with acute cerebral infarction and TIA who had undergone TCD ultrasonography were retrospectively reviewed. Their medical history, clinical manifestations, and vascular risk factors were reviewed from a stroke registry at the Chung-Ang University Hospital. ND was defined as increase of two or more National Institutes of Health Stroke Scale (NIHSS) score [22]. The NIHSS score was evaluated by a neurologist who was unaware of TCD results regularly.

Each stroke patient was examined with brain magnetic resonance imaging (MRI) and CT angiography (CTA). Cerebral small vessel disease burden was gathered from MRI and cerebral atherosclerosis from brain CTA. Old lacunes were determined by round or ovoid hypointense lesions which were encompassed by an hyperintense rim measuring < 1.5 cm in size at one of the perforating artery territories. Cerebral microbleed was defined as round or ovoid hypointense lesions appearing on susceptibility-weighted images, excluding traumatic hemorrhage or calcification lesions. Cerebral atherosclerosis score (CAS) was calculated by the sum of the degrees of stenosis of the intracranial arteries on brain CTA. Stenosis of intracranial arteries was identified at bilateral anterior/middle/posterior cerebral arteries, BA, intracranial portion of internal carotid arteries, and vertebral arteries and scored as follows: 0, no stenosis; 1, < 50% stenosis; 2, > 50% stenosis but no occlusion; and 3, occlusion.

### Transcranial Doppler ultrasonographic examination

Within 7 days of admission, TCD ultrasonography was performed by an experienced medical technician with a 2-MHz probe and Companion III (Nicolet EME, Bristol, UK). The sonographic parameters including peak systolic flow velocities (PSVs), peak diastolic velocities (PDVs), and mean flow velocities, were measured from the bilateral MCAs, BA, and other sites. All sonographic measurements of BA were performed via a transforaminal window with an insonation depth of 80–100 mm in the lying position. PI was calculated according to the Gosling formula [(PSV – PDV) / {(PSV + 2PDV) / 3}] as described in previous studies [19, 23]. All the results from TCD ultrasonography were interpreted by certified neurologists.

### Statistical analysis

All statistical analyses were performed using IBM SPSS ver. 21.0 (IBM Corp., Armonk, NY, USA) and R ver. 3.5.1 (The R Foundation for Statistical Computing, Vienna, Austria; https://www.r-project.org/; July 2, 2018). First, the patients were divided into four groups according to BA PI quartiles. The differences among the groups for categorical variables were assessed using the Fisher’s exact or Pearson chi-tests, the NIHSS and CAS was compared using the Mann-Whitney U-tests or Kruskal-Wallis tests, and the differences among the groups for continuous variables were assessed using Student t-tests or one-way analysis of variance tests. Data are expressed as means ± standard deviation for continuous variables and number (%) for categorical variables. The correlation between BA PI and MCA PI was analyzed by Pearson correlation analysis.

Second, the patients were grouped into patients with and without ND to derive factors associated with ND. The differences between the groups were assessed using the Pearson chi-tests for categorical variables and the Student t-tests for continuous variables. NIHSS and CAS were compared using Mann-Whitney U-tests. Multivariable logistic regression analyses using a forward stepwise method were performed to find independent factors related to ND with adjustments for factors derived from bivariate analysis. The results were presented as adjusted odds ratios (ORs) with 95% confidence intervals (CIs). A *P-*value of < 0.05 was regarded as statistically significant. Multivariable logistic regression analysis included the factors with *P-*value less than 0.10 from bivariable analysis.

## Results

A total of 779 consecutive patients with acute ischemic stroke and TIA were registered in the Chung-Ang University Hospital Stroke Registry during the study period. Among them, 708 patients (mean age, 68.2 ± 13.0 years; 347 female patients) who had undergone TCD ultrasonography were finally included. The mean BA PI was 0.96 ± 0.23, and the patients were categorized into four subgroups according to their BA PI values with the following cutoff points: 0.80, 0.94, and 1.10 (Table 1). As BA PI increases, mean age, right MCA PI, and the proportion of ND, females, HTN, DM, old lacunes, and white matter hyperintensity lesions also increased (Table 1). BA PI was well correlated with right MCA PI (*n =* 474, r^2^ = 0.573, *P <* 0.001) but not well with left MCA PI (*n =* 456, r^2^ = 0.0003, *P =* 0.684).

**Table 1:** Clinical characteristics of the study population according to BA PI

Characteristics	Group 1 (*n =* 178)^a)^	Group 2 (*n =* 192)^b)^	Group 3 (*n =* 219)^c)^	Group 4 (*n =* 119)^d)^	*P-*value
Age (yr)	59.7 ± 14.0	66.5 ± 12.0	72.0 ± 10.4	76.3 ± 8.7	< 0.001^***^
Female sex	73 (41.0)	90 (46.9)	119 (54.3)	65 (54.6)	0.031^*^
Neurological deterioration	19 (10.7)	20 (10.4)	28 (12.8)	25 (21.0)	0.033^*^
Hypertension	96 (53.9)	121 (63.0)	147 (67.1)	96 (80.7)	< 0.001^***^
Diabetes mellitus	39 (21.9)	64 (33.3)	87 (39.7)	49 (41.2)	0.001^**^
Current smoking	53 (29.8)	50 (26.0)	62 (28.3)	28 (23.5)	0.644
Atrial fibrillation	29 (16.3)	40 (20.8)	42 (19.2)	31 (26.1)	0.220
Previous stroke	40 (22.5)	37 (19.3)	44 (20.1)	31 (26.1)	0.497
SBP (mmHg)	144.8 ± 25.8	144.5 ± 26.6	150.4 ± 28.1	149.1 ± 26.9	0.074
Hematocrit	41.0 ± 6.3	40.8 ± 5.6	39.6 ± 5.5	40.0 ± 5.5	0.068
Leukocytes (10^9^/L)	7.80 ± 2.83	8.62 ± 6.46	8.41 ± 3.24	8.04 ± 3.67	0.157
Fasting blood glucose (mmol/L)	3.40 ± 1.51	3.70 ± 1.45	3.71 ± 1.57	3.87 ± 1.67	0.065
HbA1c (%)	5.99 ± 1.35	6.07 ± 1.32	6.37 ± 1.43	6.31 ± 1.34	0.027^*^
Total cholesterol (mmol/L)	4.81 ± 1.26	4.67 ± 1.31	4.74 ± 1.25	4.67 ± 1.30	0.680
LDL cholesterol (mmol/L)	2.81 ± 0.89	2.77 ± 0.95	2.79 ± 0.89	2.75 ± 0.90	0.943
hsCRP (mmol/L)	0.17 ± 0.57	0.19 ± 0.57	0.22 ± 0.64	0.37 ± 1.06	0.278
Homocysteine (μmol/L)	14.94 ± 7.53	14.61 ± 6.28	15.19 ± 6.20	16.77 ± 6.89	0.051
Right MCA PI (*n =* 474)	0.75 ± 0.13 (*n =* 138)	0.87 ± 0.13 (*n =* 136)	1.02 ± 0.16 (*n =* 137)	1.23 ± 0.20 (*n =* 63)	< 0.001^***^
Left MCA PI (*n =* 456)	1.26 ± 6.32 (*n =* 138)	0.86 ± 0.11 (*n =* 127)	1.03 ± 0.16 (*n =* 133)	1.21 ± 0.21 (*n =* 58)	< 0.001^***^
Cerebral microbleeds	70 (39.3)	86 (44.8)	101 (46.1)	55 (46.2)	0.520
Old lacunes	104 (58.4)	121 (63.0)	162 (74.0)	90 (75.6)	0.001^**^
CAS	2 (0–5)	3 (0–6)	4 (1–7)	4 (2–6)	0.181
WMH lesion	97 (54.5)	148 (77.1)	190 (86.8)	105 (88.2)	< 0.001^***^
NIHSS score at admission	2 (0–5)	2 (0–5)	2 (1–6)	3 (1–7)	0.079

Values are presented as means ± standard deviation, number (%), or median (interquartile range). Differences between groups were analyzed using the analysis of chi-square test and the one-way analysis of variance test. Kruskal-Wallis tests were used to compare the CAS between groups

*BA * Basilar artery, *PI P*ulsatility index, *SBP S*ystolic blood pressure, *HbA1c H*emoglobin A1c, *LDL L*ow-density lipoprotein, *hsCRP H*igh sensitivity C-reactive protein, *MCA R*ight middle cerebral artery, *CAS C*erebral atherosclerosis score, *WMH W*hite matter hyperintensity, *NIHSS *National Institutes of Health Stroke Scale

^a)^ BA PI, 0.42–0.80; ^b)^ BA PI, 0.80–0.94; ^c)^ BA PI, 0.94–1.10; ^d)^ BA PI, 1.10–2.50

^*^P < 0.05, ^**^P < 0.01, ^***^P < 0.001

ND occurred in 92 patients (13.0%). Comparison between patients with and without ND revealed that ND was associated with older age, female, higher systolic BP (SBP), BA PI, CAS and NIHSS at admission, lower serum homocysteine level, not-current smoking state, presence of white matter hyperintensity lesions, and atrial fibrillation (Table 2). BA PI was higher in patients with ND (1.02 ± 0.26) than in neurologically stable patients (0.95 ± 0.22). Multivariable logistic regression model including age, female sex, history of HTN, DM, atrial fibrillation, current smoking status, SBP, serum homocysteine, old lacunes on brain MRI and CAS derived from brain CTA revealed that the higher BA PI (OR, 3.28; 95% CI, 1.07–10.17; *P =* 0.038) (Table 3) and highest BA PI quartile was independently associated with ND (OR, 2.39; 95% CI, 1.10–5.25; *P =* 0.028) (Table 3).

**Table 2:** Comparison among patients with and without neurological deterioration ^a)^

Variable	Neurological deterioration (−) (*n =* 616)	Neurological deterioration (+) (*n =* 92)	*P-*value
Age (yr)	67.7 ± 13.2	71.2 ± 11.2	0.016^*^
Female sex	293 (47.6)	54 (58.7)	0.060^**^
Hypertension	396 (64.3)	64 (69.6)	0.383
Diabetes mellitus	206 (33.4)	33 (35.9)	0.733
Current smoking	177 (28.7)	16 (17.4)	0.031^*^
Atrial fibrillation	113 (18.3)	29 (31.5)	0.005^*^
Previous stroke	129 (20.9)	23 (25.0)	0.454
SBP (mmHg)	146.3 ± 27.1	153.2 ± 25.7	0.023^*^
Hematocrit	40.4 ± 5.9	40.4 ± 5.0	0.954
Leukocytes (10^9^/L)	8.21 ± 4.43	8.51 ± 3.75	0.485
Fasting blood glucose (mmol/L)	3.65 ± 1.59	3.70 ± 1.19	0.704
HbA1c (%)	6.20 ± 1.42	6.07 ± 1.04	0.278
Total cholesterol (mmol/L)	4.74 ± 1.30	4.67 ± 1.07	0.565
LDL cholesterol (mmol/L)	2.78 ± 0.92	2.77 ± 0.84	0.898
hsCRP (mmol/L)	0.22 ± 0.67	0.25 ± 0.85	0.725
Homocysteine (μmol/L)	15.40 ± 6.92	14.16 ± 5.09	0.043^*^
Basilar artery PI	0.95 ± 0.22	1.02 ± 0.26	0.014^*^
Right MCA PI (*n =* 474)	0.92 ± 0.21 (*n =* 422)	0.97 ± 0.27 (*n =* 52)	0.285
Left MCA PI (*n =* 456)	1.09 ± 3.70 (*n =* 406)	0.96 ± 0.24 (*n =* 50)	0.170
Cerebral microbleeds	267 (43.3)	45 (48.9)	0.373
Old lacunes	416 (67.5)	61 (66.3)	0.908
CAS	3 (0–6)	5 (2–8)	0.002^***^
WMH lesion	461 (74.8)	79 (85.9)	0.029^*^
NIHSS score at admission	2 (0–5)	6 (3–9)	< 0.001^***^

Values are presented as means ± standard deviation, number (%), or median (interquartile range). Differences between groups were analyzed using the analysis of chi-square test and one-way analysis of variance test. Mann-Whitney U-tests were used for comparing NIHSS and CAS between groups

*SBP S*ystolic blood pressure, *HbA1c H*emoglobin A1c, *LDL L*ow-density lipoprotein, *hsCRP H*igh sensitivity C-reactive protein, *PI P*ulsatility index, *MCA R*ight middle cerebral artery, *CAS * Cerebral atherosclerosis score, *WMH W*hite matter hyperintensity, *NIHSS *National Institutes of Health Stroke Scale

^a)^ Univariably significant with a *P-*value of ≤0.10 and considered in a multivariable model

^*^P < 0.05, ^**^P < 0.1, ^***^P < 0.01

**Table 3:** Logistic regression analysis for the determinants of early neurological deterioration

Variable	Bivariable analyses		Multivariable analyses	
	OR (95% CI)	*P-*value	Adjusted OR (95% CI)^a)^	*P-*value
BA PI	3.59 (1.45–8.81)	0.005^*^	3.28 (1.07–10.17)	0.038^**^
BA PI, quartile^b)^				
Q1 (0.42–0.80)	1		1	
Q2 (0.80–0.94)	0.97 (0.50–1.89)	0.935	0.93 (0.45–1.90)	0.841
Q3 (0.94–1.10)	1.23 (0.66–2.28)	0.518	1.05 (0.52–2.15)	0.890
Q4 (1.10–2.50)	2.22 (1.16–4.26)	0.016^**^	2.39 (1.10–5.25)	0.028^**^

BA PI measured by transcranial Doppler ultrasonography

*OR O*dds ratio, *CI C*onfidence interval, *BA PI B*asilar artery pulsatility index

^a)^ Adjusted by age, sex, history of atrial fibrillation, current smoking status, National Institutes of Health Stroke Scale at admission, systolic blood pressure, serum homocysteine level, cerebral atherosclerosis score, and white matter hyperintensity lesion. ^b)^ Group was divided into quartiles based on BA PI

^*^P < 0.01, ^**^P < 0.05

## Discussion

In this study including 708 acute stroke patients who had undergone brain MRI, CTA, and TCD ultrasonographic examination, ND occurred in 92 patients (13%) and the proportion of patients with ND was the highest in the highest BA PI quartile group. Multivariable logistic regression analysis including clinical and imaging variables showed that BA PI is an independent factor associated with ND. Although right MCA PI was well correlated with BA PI, their detection was not possible owing to poor temporal windows among more than one third of the included patients.

Exaggerated pulsatile cerebral blood flow can result in cerebrovascular endothelial failure, blood-brain barrier disruption, perfusion decrease during diastolic phase, and increase in endothelial shear stress [11, 13, 16, 17, 24]. Several studies have demonstrated that elevated PI is linked with an inverse nonlinear relationship of cerebral perfusion pressure and linear relationship of intracranial pressure as well as with an increased cerebral vascular resistance and cerebral small vessel disease burden [22, 25, 26]. Additionally, it has been demonstrated that the MCA PI and BA PI increase in patients with HTN [27, 28]. Our study also showed an increasing tendency of old lacunes and white matter hyperintensities according to the BA PI quartile, suggesting that small vessel disease burden is related to cerebral arterial stiffness.

Early ND with ischemia progression can occur due to decreased cerebral blood flow from parent artery or lack of collateral circulation. Previous study showed that higher pulsatility of MCA was associated with progression in lacunar infarction [29]. Since PI measured by transcranial Doppler sonography might reflect downstream arterial resistance and vascular perfusion status, elevated PI could be a possible indicator of stroke progression [29].

Elevated MCA PI is reported to be associated with deterioration of lacunar cerebral infarction [17]. Consistent with a previous report, MCA blood flow could not be detected in this study owing to poor acoustical temporal windows in more than 30% of patients [30]. Contrary to MCA PI, BA PI can be measured irrespective of temporal bone windows. Previous study showed that BA PI is well correlated with MCA PI among the lacunar stroke patients with DM [31]. Another previous study reported that BA PI increased earlier than MCA PI in patients with microangiopathy complicated with DM because vessels in the posterior cerebral circulation have fewer adrenergic neurons which regulate vascular tone in response to stimulations other than those in the anterior cerebral circulation [20].

The study has several limitations. First, the cross-sectional design of our analyses limits our ability to determine a causal relationship between BA PI and ND. Second, BA PI was only measured at admission, which yielded no data regarding the temporal change during acute cerebral infarction. Third, this study was performed in a single hospital in Seoul, Korea; therefore, more studies are required to generalize our findings. The strength of this study is that we constructed a multivariable logistic model including clinical, laboratory, and imaging variables of brain MRI and CTA and confirmed the independent association between BA PI and ND.

## Conclusions

This study showed that high BA PI could be associated with ND in acute stroke patients. BA PI could help to predict ND among stroke patients, and proactive management strategy for at-risk stroke patients are required to prevent stroke progression.

## Supplementary Information

**Additional file 1.**

## Data Availability

Not applicable.

## Abbreviations

BA: Basilar artery
BP: Blood pressure
CAS: Cerebral atherosclerosis score
CI: Confidence interval
CT: Computed tomography
CTA: Computed tomography angiography
DM: Diabetes mellitus
HTN: Hypertension
MCA: Middle cerebral artery
MRI: Magnetic resonance imaging
ND: Neurological deterioration
NIHSS: National Institutes of Health Stroke Scale
OR: Odds ratio
PDV: Peak diastolic velocity
PI: Pulsatility index
PSV: Peak systolic flow velocity
SBP: Systolic blood pressure
TCD: Transcranial Doppler
TIA: Transient ischemic attack
