# Supplementary material for: Increased pulsatility index of the basilar artery is a risk factor for neurological deterioration after stroke: a case control study
Source: Clin Hypertens. 2022 Aug 15;28:27. doi: 10.1186/s40885-022-00210-9 (PMC9377089; doi:10.1186/s40885-022-00210-9)
Supplement: Supplementary file 1 — Additional file 1. [file 40885_2022_210_MOESM1_ESM.docx]

**Increased pulsatility index of the basilar artery is a risk factor for neurological deterioration after stroke: a case control study**

**Il-Han Yoo^1^, Jeong-Min Kim^2*^, Su-Hyun Han^3^, Jaiyoung Ryu^4^, Keun-Hwa Jung^2^ and Kwang-Yeol Park^3^**

^1^Department of Neurology, Nowon Eulji Medical Center, Eulji University School of Medicine, Seoul, Republic of Korea

^2^Department of Neurology, Seoul National University Hospital, Seoul National University College of Medicine, Seoul, Republic of Korea

^3^Department of Neurology, Chung-Ang University Hospital, Chung-Ang University College of Medicine, Seoul, Republic of Korea

^4^Department of Mechanical Engineering, Chung-Ang University, Seoul, Republic of Korea

***Correspondence:** bellokim1@gmail.com

**ORCID**

Il-Han Yoo: https://orcid.org/0000-0003-2793-1617

Jeong-Min Kim: https://orcid.org/0000-0001-7213-5527

Su-Hyun Han: https://orcid.org/0000-0002-3084-6985

Jaiyoung Ryu: https://orcid.org/0000-0001-7846-4700

Keun-Hwa Jung: https://orcid.org/0000-0003-1433-8005

Kwang-Yeol Park: https://orcid.org/0000-0003-4570-3538

**Abstract**

**Background:** Higher pulsatility of the middle cerebral artery (MCA) is known to be associated with stroke progression. We investigated whether pulsatility index (PI) of the basilar artery (BA) can predict neurological deterioration (ND) after acute cerebral infarction.

**Methods:** A total of 708 consecutive patients with acute ischemic stroke who had undergone transcranial Doppler (TCD) ultrasonography were included. ND was defined as an increase in the National Institutes of Health Stroke Scale scores by two or more points after admission. The patients were categorized into quartiles according to BA PI. Multivariable logistic regression analysis was performed to examine whether BA PI is independently associated with ND.

**Results:** BA PI was well correlated with the right (n = 474, r^2^ = 0.573, P < 0.001) by Pearson correlation analysis although MCA could not be measured from right MCA (n = 234, 33.05%) and left MCA (n = 252, 35.59%) by TCD owing to insufficient temporal bone window. Multivariable logistic regression analysis including age, sex, cerebral atherosclerosis burden, National Institutes of Health Stroke Scale at admission, and the proportion of patients with current smoking status, hypertension, diabetes mellitus, atrial fibrillation revealed that the higher BA PI (odds ratio, 3.28; confidence interval, 1.07–10.17; P = 0.038) was independently associated with ND.

**Conclusions:** BA PI, which would be identified regardless of temporal window, could predict ND among acute stroke patients.

**Keywords:** Basilar artery, Stroke, Transcranial Doppler sonography­­­­­­­­­, Neurological deterioration

**Background**

Neurological deterioration (ND) occurs in 10% to 58% adult stroke patients and results in poor prognosis and mortality [1–5]. Several factors are known to be associated with ND, such as old age, diabetes mellitus (DM), hypertension (HTN), smoking habit, coronary heart disease, the size of low-density lesions as observed on initial computed tomography (CT), change in the flow velocity of middle cerebral artery (MCA), impaired cerebral hemodynamic reserve, blood glucose level, proinflammatory cytokine level, and blood pressure (BP) [5–10]. From a mechanistic perspective, failed intracranial collateral blood flow or elevated intracranial pressure may lead to decreased cerebral perfusion, thereby causing ND [2].

Because the stiffness of large arteries is linked with various cerebral small vessel disease phenotypes including cerebral microbleeds, white matter hyperintensities, and lacunar cerebral infarction, it may be plausible that increased cerebral arterial stiffness is associated with ND after stroke [11–17]. The pulsatility index (PI) of intracranial cerebral arteries, as measured by transcranial Doppler (TCD) ultrasonography, is known to reflect the resistance of downstream arteries and compliance of large cerebral arteries [17–20]. Recent study reported that elevated MCA PI is independently associated with ND among lacunar stroke patients [17]. However, in 18% of patients with acute ischemic stroke or transient ischemic attack (TIA), MCA PI is unobtainable due to poor acoustical temporal bone window; It is known that a poor acoustical temporal bone window is more common in not only elderly patients, but also female patients and those with thick skull [21].

The basilar artery (BA) PI can be measured through the transforaminal approach, which the transducer is placed just below the occipital protuberance and directed towards the nasal bridge. However, the clinical significance of BA PI among stroke patients has not been appreciated yet. We investigated whether BA PI can predict ND after acute stroke.

**Methods**

**Patients and evaluation**

From January 2014 to December 2015, consecutive patients with acute cerebral infarction and TIA who had undergone TCD ultrasonography were retrospectively reviewed. Their medical history, clinical manifestations, and vascular risk factors were reviewed from a stroke registry at the Chung-Ang University Hospital. ND was defined as increase of two or more National Institutes of Health Stroke Scale (NIHSS) score [22]. The NIHSS score was evaluated by a neurologist who was unaware of TCD results regularly.

Each stroke patient was examined with brain magnetic resonance imaging (MRI) and CT angiography (CTA). Cerebral small vessel disease burden was gathered from MRI and cerebral atherosclerosis from brain CTA. Old lacunes were determined by round or ovoid hypointense lesions which were encompassed by an hyperintense rim measuring <1.5 cm in size at one of the perforating artery territories. Cerebral microbleed was defined as round or ovoid hypointense lesions appearing on susceptibility-weighted images, excluding traumatic hemorrhage or calcification lesions. Cerebral atherosclerosis score (CAS) was calculated by the sum of the degrees of stenosis of the intracranial arteries on brain CTA. Stenosis of intracranial arteries was identified at bilateral anterior/middle/posterior cerebral arteries, BA, intracranial portion of internal carotid arteries, and vertebral arteries and scored as follows: 0, no stenosis; 1, <50% stenosis; 2, >50% stenosis but no occlusion; and 3, occlusion.

**Transcranial Doppler ultrasonographic examination**

Within 7 days of admission, TCD ultrasonography was performed by an experienced medical technician with a 2-MHz probe and Companion III (Nicolet EME, Bristol, UK). The sonographic parameters including peak systolic flow velocities (PSVs), peak diastolic velocities (PDVs), and mean flow velocities, were measured from the bilateral MCAs, BA, and other sites. All sonographic measurements of BA were performed via a transforaminal window with an insonation depth of 80–100 mm in the lying position. PI was calculated according to the Gosling formula [(PSV – PDV) / {(PSV + 2PDV) / 3}] as described in previous studies [19, 23]. All the results from TCD ultrasonography were interpreted by certified neurologists.

**Statistical analysis**

All statistical analyses were performed using IBM SPSS ver. 21.0 (IBM Corp., Armonk, NY, USA) and R ver. 3.5.1 (The R Foundation for Statistical Computing, Vienna, Austria; https://www.r-project.org/; July 2, 2018). First, the patients were divided into four groups according to BA PI quartiles. The differences among the groups for categorical variables were assessed using the Fisher’s exact or Pearson chi-tests, the NIHSS and CAS was compared using the Mann-Whitney U-tests or Kruskal-Wallis tests, and the differences among the groups for continuous variables were assessed using Student t-tests or one-way analysis of variance tests. Data are expressed as means ± standard deviation for continuous variables and number (%) for categorical variables. The correlation between BA PI and MCA PI was analyzed by Pearson correlation analysis.

Second, the patients were grouped into patients with and without ND to derive factors associated with ND. The differences between the groups were assessed using the Pearson chi-tests for categorical variables and the Student t-tests for continuous variables. NIHSS and CAS were compared using Mann-Whitney U-tests. Multivariable logistic regression analyses using a forward stepwise method were performed to find independent factors related to ND with adjustments for factors derived from bivariate analysis. The results were presented as adjusted odds ratios (ORs) with 95% confidence intervals (CIs). A P-value of <0.05 was regarded as statistically significant. Multivariable logistic regression analysis included the factors with P-value less than 0.10 from bivariable analysis.

**Results**

A total of 779 consecutive patients with acute ischemic stroke and TIA were registered in the Chung-Ang University Hospital Stroke Registry during the study period. Among them, 708 patients (mean age, 68.2 ± 13.0 years; 347 female patients) who had undergone TCD ultrasonography were finally included. The mean BA PI was 0.96 ± 0.23, and the patients were categorized into four subgroups according to their BA PI values with the following cutoff points: 0.80, 0.94, and 1.10 (Table 1). As BA PI increases, mean age, right MCA PI, and the proportion of ND, females, HTN, DM, old lacunes, and white matter hyperintensity lesions also increased (Table 1). BA PI was well correlated with right MCA PI (n = 474, r^2^ = 0.573, P < 0.001) but not well with left MCA PI (n = 456, r^2^ = 0.0003, P = 0.684).

ND occurred in 92 patients (13.0%). Comparison between patients with and without ND revealed that ND was associated with older age, female, higher systolic BP (SBP), BA PI, CAS and NIHSS at admission, lower serum homocysteine level, not-current smoking state, presence of white matter hyperintensity lesions, and atrial fibrillation (Table 2). BA PI was higher in patients with ND (1.02 ± 0.26) than in neurologically stable patients (0.95 ± 0.22). Multivariable logistic regression model including age, female sex, history of HTN, DM, atrial fibrillation, current smoking status, SBP, serum homocysteine, old lacunes on brain MRI and CAS derived from brain CTA revealed that the higher BA PI (OR, 3.28; 95% CI, 1.07–10.17; P = 0.038) (Table 3) and highest BA PI quartile was independently associated with ND (OR, 2.39; 95% CI, 1.10–5.25; P = 0.028) (Table 3).

**Discussion**

In this study including 708 acute stroke patients who had undergone brain MRI, CTA, and TCD ultrasonographic examination, ND occurred in 92 patients (13%) and the proportion of patients with ND was the highest in the highest BA PI quartile group. Multivariable logistic regression analysis including clinical and imaging variables showed that BA PI is an independent factor associated with ND. Although right MCA PI was well correlated with BA PI, their detection was not possible owing to poor temporal windows among more than one third of the included patients.

Exaggerated pulsatile cerebral blood flow can result in cerebrovascular endothelial failure, blood-brain barrier disruption, perfusion decrease during diastolic phase, and increase in endothelial shear stress [11, 13, 16, 17, 24]. Several studies have demonstrated that elevated PI is linked with an inverse nonlinear relationship of cerebral perfusion pressure and linear relationship of intracranial pressure as well as with an increased cerebral vascular resistance and cerebral small vessel disease burden [22, 25, 26]. Additionally, it has been demonstrated that the MCA PI and BA PI increase in patients with HTN [27, 28]. Our study also showed an increasing tendency of old lacunes and white matter hyperintensities according to the BA PI quartile, suggesting that small vessel disease burden is related to cerebral arterial stiffness.

Early ND with ischemia progression can occur due to decreased cerebral blood flow from parent artery or lack of collateral circulation. Previous study showed that higher pulsatility of MCA was associated with progression in lacunar infarction [29]. Since PI measured by transcranial Doppler sonography might reflect downstream arterial resistance and vascular perfusion status, elevated PI could be a possible indicator of stroke progression [29].

Elevated MCA PI is reported to be associated with deterioration of lacunar cerebral infarction [17]. Consistent with a previous report, MCA blood flow could not be detected in this study owing to poor acoustical temporal windows in more than 30% of patients [30]. Contrary to MCA PI, BA PI can be measured irrespective of temporal bone windows. Previous study showed that BA PI is well correlated with MCA PI among the lacunar stroke patients with DM [31]. Another previous study reported that BA PI increased earlier than MCA PI in patients with microangiopathy complicated with DM because vessels in the posterior cerebral circulation have fewer adrenergic neurons which regulate vascular tone in response to stimulations other than those in the anterior cerebral circulation [20].

The study has several limitations. First, the cross-sectional design of our analyses limits our ability to determine a causal relationship between BA PI and ND. Second, BA PI was only measured at admission, which yielded no data regarding the temporal change during acute cerebral infarction. Third, this study was performed in a single hospital in Seoul, Korea; therefore, more studies are required to generalize our findings. The strength of this study is that we constructed a multivariable logistic model including clinical, laboratory, and imaging variables of brain MRI and CTA and confirmed the independent association between BA PI and ND.

**Conclusions**

This study showed that high BA PI could be associated with ND in acute stroke patients. BA PI could help to predict ND among stroke patients, and proactive management strategy for at-risk stroke patients are required to prevent stroke progression.

**Abbreviations**

BA: Basilar artery; BP: Blood pressure; CAS: Cerebral atherosclerosis score; CI: Confidence interval; CT: Computed tomography; CTA: Computed tomography angiography; DM: Diabetes mellitus; HTN: Hypertension; MCA: Middle cerebral artery; MRI: Magnetic resonance imaging; ND: Neurological deterioration; NIHSS: National Institutes of Health Stroke Scale; OR: Odds ratio; PDV: Peak diastolic velocity; PI: Pulsatility index; PSV: Peak systolic flow velocity; SBP: Systolic blood pressure; TCD: Transcranial Doppler; TIA: Transient ischemic attack

**Declarations**

**Ethical approval and consent to participate**

This study was reviewed and approved by the Institutional Review Board of Chung-Ang University Hospital (C2013110) and was conducted in accordance with the principles of the Declaration of Helsinki.

**Consent for publication**

Not applicable.

**Availability of data and materials**

Not applicable.

**Competing interests**

The authors declare that they have no competing interests.

**Acknowledgements**

Not applicable.

**Funding**

The work was supported by the Basic Science Research Program through the National Research Foundation of Korea (NRF) funded by the Ministry of Education (NRF-2017R1D1A1B03029909, NRF-2019R1F1A1059455) and by the Korean Society of Hypertension (2019). The funding has no role in design, collection, analysis, or interpretation of data; in the writing of the manuscript; and in the decision to submit the manuscript for publication.

**Authors' contributions**

IHY analyzed the data, wrote and revised the manuscript. JMK and KYP conceptualized and design the study, and revised the manuscript. SHH and JR analyzed the data. All the authors have made substantial contributions so as to qualify for the authorship, and have read and approved the submission of the manuscript.

**References**

1. Helleberg BH, Ellekjaer H, Indredavik B. Outcomes after early neurological deterioration and transitory deterioration in acute ischemic stroke patients. Cerebrovasc Dis. 2016;42:378–86.

2. Thanvi B, Treadwell S, Robinson T. Early neurological deterioration in acute ischaemic stroke: predictors, mechanisms and management. Postgrad Med J. 2008;84:412–7.

3. Weimar C, Mieck T, Buchthal J, Ehrenfeld CE, Schmid E, Diener HC, et al. Neurologic worsening during the acute phase of ischemic stroke. Arch Neurol. 2005;62:393–7.

4. Caplan LR. Worsening in ischemic stroke patients: is it time for a new strategy? Stroke. 2002;33:1443–5.

5. Toni D, Fiorelli M, Gentile M, Bastianello S, Sacchetti ML, Argentino C, et al. Progressing neurological deficit secondary to acute ischemic stroke. A study on predictability, pathogenesis, and prognosis. Arch Neurol. 1995;52:670–5.

6. Cuadrado-Godia E, Jimena S, Ois A, Rodríguez-Campello A, Giralt-Steinhauer E, Soriano-Tarraga C, et al. Factors associated with early outcome in patients with large-vessel carotid strokes. J Neurol Neurosurg Psychiatry. 2013;84:305–9.

7. Wakugawa Y, Kiyohara Y, Tanizaki Y, Kubo M, Ninomiya T, Hata J, et al. C-reactive protein and risk of first-ever ischemic and hemorrhagic stroke in a general Japanese population: the Hisayama Study. Stroke. 2006;37:27–32.

8. Dávalos A, Toni D, Iweins F, Lesaffre E, Bastianello S, Castillo J. Neurological deterioration in acute ischemic stroke: potential predictors and associated factors in the European cooperative acute stroke study (ECASS) I. Stroke. 1999;30:2631–6.

9. Baizabal-Carvallo JF, Alonso-Juarez M, Samson Y. Clinical deterioration following middle cerebral artery hemodynamic changes after intravenous thrombolysis for acute ischemic stroke. J Stroke Cerebrovasc Dis. 2014;23:254–8.

10. Dávalos A, Castillo J, Marrugat J, Fernandez-Real JM, Armengou A, Cacabelos P, et al. Body iron stores and early neurologic deterioration in acute cerebral infarction. Neurology. 2000;54:1568–74.

11. Wohlfahrt P, Krajcoviechova A, Jozifova M, Mayer O, Vanek J, Filipovsky J, et al. Large artery stiffness and carotid flow pulsatility in stroke survivors. J Hypertens. 2014;32:1097–103.

12. van Elderen SG, Brandts A, Westenberg JJ, van der Grond J, Tamsma JT, van Buchem MA, et al. Aortic stiffness is associated with cardiac function and cerebral small vessel disease in patients with type 1 diabetes mellitus: assessment by magnetic resonance imaging. Eur Radiol. 2010;20:1132–8.

13. Henskens LH, Kroon AA, van Oostenbrugge RJ, Gronenschild EH, Fuss-Lejeune MM, Hofman PA, et al. Increased aortic pulse wave velocity is associated with silent cerebral small-vessel disease in hypertensive patients. Hypertension. 2008;52:1120–6.

14. Tsao CW, Seshadri S, Beiser AS, Westwood AJ, Decarli C, Au R, et al. Relations of arterial stiffness and endothelial function to brain aging in the community. Neurology. 2013;81:984–91.

15. Brandts A, van Elderen SG, Westenberg JJ, van der Grond J, van Buchem MA, Huisman MV, et al. Association of aortic arch pulse wave velocity with left ventricular mass and lacunar brain infarcts in hypertensive patients: assessment with MR imaging. Radiology. 2009;253:681–8.

16. Seo WK, Lee JM, Park MH, Park KW, Lee DH. Cerebral microbleeds are independently associated with arterial stiffness in stroke patients. Cerebrovasc Dis. 2008;26:618–23.

17. Kidwell CS, el-Saden S, Livshits Z, Martin NA, Glenn TC, Saver JL. Transcranial Doppler pulsatility indices as a measure of diffuse small-vessel disease. J Neuroimaging. 2001;11:229–35.

18. Giller CA, Hodges K, Batjer HH. Transcranial Doppler pulsatility in vasodilation and stenosis. J Neurosurg. 1990;72:901–6.

19. de Riva N, Budohoski KP, Smielewski P, Kasprowicz M, Zweifel C, Steiner LA, et al. Transcranial Doppler pulsatility index: what it is and what it isn't. Neurocrit Care. 2012;17:58–66.

20. Lee KY, Sohn YH, Baik JS, Kim GW, Kim JS. Arterial pulsatility as an index of cerebral microangiopathy in diabetes. Stroke. 2000;31:1111–5.

21. Wijnhoud AD, Franckena M, van der Lugt A, Koudstaal PJ, Dippel ED. Inadequate acoustical temporal bone window in patients with a transient ischemic attack or minor stroke: role of skull thickness and bone density. Ultrasound Med Biol. 2008;34:923–9.

22. Kwan J, Hand P. Early neurological deterioration in acute stroke: clinical characteristics and impact on outcome. QJM. 2006;99:625–33.

23. Blanco P, Abdo-Cuza A. Transcranial Doppler ultrasound in neurocritical care. J Ultrasound. 2018;21:1-16.

24. Shi Y, Thrippleton MJ, Marshall I, Wardlaw JM. Intracranial pulsatility in patients with cerebral small vessel disease: a systematic review. Clin Sci (Lond). 2018;132:157–71.

25. Calviello LA, de Riva N, Donnelly J, Czosnyka M, Smielewski P, Menon DK, et al. Relationship between brain pulsatility and cerebral perfusion pressure: replicated validation using different drivers of CPP change. Neurocrit Care. 2017;27:392–400.

26. Restrepo L, Razumovsky AY, Ziai W, Barker PB, Beauchamp NJ, Wityk RJ. Transcranial Doppler markers of diffusion-perfusion mismatch. J Neuroimaging. 2003;13:34–42.

27. Cho SJ, Sohn YH, Kim GW, Kim JS. Blood flow velocity changes in the middle cerebral artery as an index of the chronicity of hypertension. J Neurol Sci. 1997;150:77–80.

28. Jeong HT, Kim DS, Kang KW, Nam YT, Oh JE, Cho EK. Factors affecting basilar artery pulsatility index on transcranial Doppler. Korean J Clin Lab Sci. 2018;50:477–83.

29. Lee KJ, Jung KH, Park CY, Kim JM, Lee ST, Chu K, et al. Increased arterial pulsatility and progression of single subcortical infarction. Eur Radiol. 2017;27:899–906.

30. Sarkar S, Ghosh S, Ghosh SK, Collier A. Role of transcranial Doppler ultrasonography in stroke. Postgrad Med J. 2007;83:683–9.

31. Lee KO, Park JH, Choi YC, Han SW, Nam HS, Heo JH, et al. Increased pulsatility index in acute lacunar infarction with type II diabetes. J Korean Neurol Assoc. 2005;23:457–62.

**Table 1.** Clinical characteristics of the study population according to BA PI

| Characteristics | Group 1  (n = 178)^a)^ | Group 2  (n = 192)^b)^ | Group 3  (n = 219)^c)^ | Group 4  (n = 119)^d)^ | P-value |
| --- | --- | --- | --- | --- | --- |
| Age (yr) | 59.7 ± 14.0 | 66.5 ± 12.0 | 72.0 ± 10.4 | 76.3 ± 8.7 | <0.001^***^ |
| Female sex | 73 (41.0) | 90 (46.9) | 119 (54.3) | 65 (54.6) | 0.031^*^ |
| Neurological deterioration | 19 (10.7) | 20 (10.4) | 28 (12.8) | 25 (21.0) | 0.033^*^ |
| Hypertension | 96 (53.9) | 121 (63.0) | 147 (67.1) | 96 (80.7) | <0.001^***^ |
| Diabetes mellitus | 39 (21.9) | 64 (33.3) | 87 (39.7) | 49 (41.2) | 0.001^**^ |
| Current smoking | 53 (29.8) | 50 (26.0) | 62 (28.3) | 28 (23.5) | 0.644 |
| Atrial fibrillation | 29 (16.3) | 40 (20.8) | 42 (19.2) | 31 (26.1) | 0.220 |
| Previous stroke | 40 (22.5) | 37 (19.3) | 44 (20.1) | 31 (26.1) | 0.497 |
| SBP (mmHg) | 144.8 ± 25.8 | 144.5 ± 26.6 | 150.4 ± 28.1 | 149.1 ± 26.9 | 0.074 |
| Hematocrit | 41.0 ± 6.3 | 40.8 ± 5.6 | 39.6 ± 5.5 | 40.0 ± 5.5 | 0.068 |
| Leukocytes (10^9^/L) | 7.80 ± 2.83 | 8.62 ± 6.46 | 8.41 ± 3.24 | 8.04 ± 3.67 | 0.157 |
| Fasting blood glucose (mmol/L) | 3.40 ± 1.51 | 3.70 ± 1.45 | 3.71 ± 1.57 | 3.87 ± 1.67 | 0.065 |
| HbA1c (%) | 5.99 ± 1.35 | 6.07 ± 1.32 | 6.37 ± 1.43 | 6.31 ± 1.34 | 0.027^*^ |
| Total cholesterol (mmol/L) | 4.81 ± 1.26 | 4.67 ± 1.31 | 4.74 ± 1.25 | 4.67 ± 1.30 | 0.680 |
| LDL cholesterol (mmol/L) | 2.81 ± 0.89 | 2.77 ± 0.95 | 2.79 ± 0.89 | 2.75 ± 0.90 | 0.943 |
| hsCRP (mmol/L) | 0.17 ± 0.57 | 0.19 ± 0.57 | 0.22 ± 0.64 | 0.37 ± 1.06 | 0.278 |
| Homocysteine (μmol/L) | 14.94 ± 7.53 | 14.61 ± 6.28 | 15.19 ± 6.20 | 16.77 ± 6.89 | 0.051 |
| Right MCA PI (n = 474) | 0.75 ± 0.13  (n = 138) | 0.87 ± 0.13  (n = 136) | 1.02 ± 0.16  (n = 137) | 1.23 ± 0.20  (n = 63) | <0.001^***^ |
| Left MCA PI (n = 456) | 1.26 ± 6.32  (n = 138) | 0.86 ± 0.11  (n = 127) | 1.03 ± 0.16  (n = 133) | 1.21 ± 0.21  (n = 58) | <0.001^***^ |
| Cerebral microbleeds | 70 (39.3) | 86 (44.8) | 101 (46.1) | 55 (46.2) | 0.520 |
| Old lacunes | 104 (58.4) | 121 (63.0) | 162 (74.0) | 90 (75.6) | 0.001^**^ |
| CAS | 2 (0–5) | 3 (0–6) | 4 (1–7) | 4 (2–6) | 0.181 |
| WMH lesion | 97 (54.5) | 148 (77.1) | 190 (86.8) | 105 (88.2) | <0.001^***^ |
| NIHSS score at admission | 2 (0–5) | 2 (0–5) | 2 (1–6) | 3 (1–7) | 0.079 |

Values are presented as means ± standard deviation, number (%), or median (interquartile range).

Differences between groups were analyzed using the analysis of chi-square test and the one-way analysis of variance test. Kruskal-Wallis tests were used to compare the CAS between groups.

BA, basilar artery; PI, pulsatility index; SBP, systolic blood pressure; HbA1c, hemoglobin A1c; LDL, low-density lipoprotein; hsCRP, high sensitivity C-reactive protein; MCA, right middle cerebral artery; CAS, cerebral atherosclerosis score; WMH, white matter hyperintensity; NIHSS, National Institutes of Health Stroke Scale.

^a)^BA PI, 0.42–0.80; ^b)^BA PI, 0.80–0.94; ^c)^BA PI, 0.94–1.10; ^d)^BA PI, 1.10–2.50.

^*^P < 0.05, ^**^P < 0.01, ^***^P < 0.001.

**Table 2**. Comparison among patients with and without neurological deterioration ^a)^

| Variable | Neurological deterioration (−) (n = 616) | Neurological deterioration (+) (n = 92) | P-value |
| --- | --- | --- | --- |
| Age (yr) | 67.7 ± 13.2 | 71.2 ± 11.2 | 0.016^*^ |
| Female sex | 293 (47.6) | 54 (58.7) | 0.060^**^ |
| Hypertension | 396 (64.3) | 64 (69.6) | 0.383 |
| Diabetes mellitus | 206 (33.4) | 33 (35.9) | 0.733 |
| Current smoking | 177 (28.7) | 16 (17.4) | 0.031^*^ |
| Atrial fibrillation | 113 (18.3) | 29 (31.5) | 0.005^*^ |
| Previous stroke | 129 (20.9) | 23 (25.0) | 0.454 |
| SBP (mmHg) | 146.3 ± 27.1 | 153.2 ± 25.7 | 0.023^*^ |
| Hematocrit | 40.4 ± 5.9 | 40.4 ± 5.0 | 0.954 |
| Leukocytes (10^9^/L) | 8.21 ± 4.43 | 8.51 ± 3.75 | 0.485 |
| Fasting blood glucose (mmol/L) | 3.65 ± 1.59 | 3.70 ± 1.19 | 0.704 |
| HbA1c (%) | 6.20 ± 1.42 | 6.07 ± 1.04 | 0.278 |
| Total cholesterol (mmol/L) | 4.74 ± 1.30 | 4.67 ± 1.07 | 0.565 |
| LDL cholesterol (mmol/L) | 2.78 ± 0.92 | 2.77 ± 0.84 | 0.898 |
| hsCRP (mmol/L) | 0.22 ± 0.67 | 0.25 ± 0.85 | 0.725 |
| Homocysteine (μmol/L) | 15.40 ± 6.92 | 14.16 ± 5.09 | 0.043^*^ |
| Basilar artery PI | 0.95 ± 0.22 | 1.02 ± 0.26 | 0.014^*^ |
| Right MCA PI (n = 474) | 0.92 ± 0.21  (n = 422) | 0.97 ± 0.27  (n = 52) | 0.285 |
| Left MCA PI (n = 456) | 1.09 ± 3.70  (n = 406) | 0.96 ± 0.24  (n = 50) | 0.170 |
| Cerebral microbleeds | 267 (43.3) | 45 (48.9) | 0.373 |
| Old lacunes | 416 (67.5) | 61 (66.3) | 0.908 |
| CAS | 3 (0–6) | 5 (2–8) | 0.002^***^ |
| WMH lesion | 461 (74.8) | 79 (85.9) | 0.029^*^ |
| NIHSS score at admission | 2 (0–5) | 6 (3–9) | <0.001^***^ |

Values are presented as means ± standard deviation, number (%), or median (interquartile range). Differences between groups were analyzed using the analysis of chi-square test and one-way analysis of variance test. Mann-Whitney U-tests were used for comparing NIHSS and CAS between groups.

SBP, systolic blood pressure; HbA1c, hemoglobin A1c; LDL, low-density lipoprotein; hsCRP, high sensitivity C-reactive protein; PI, pulsatility index; MCA, right middle cerebral artery; CAS, cerebral atherosclerosis score; WMH, white matter hyperintensity, NIHSS, National Institutes of Health Stroke Scale.

^a)^Univariably significant with a P-value of ≤0.10 and considered in a multivariable model.

^*^P < 0.05, ^**^P < 0.1, ^***^P < 0.01.

**Table 3.** Logistic regression analysis for the determinants of early neurological deterioration

| **Variable** | Bivariable analyses | | Multivariable analyses | |
| --- | --- | --- | --- | --- |
|  | OR (95% CI) | P-value | Adjusted OR (95% CI)^a)^ | P-value |
| BA PI | 3.59 (1.45–8.81) | 0.005**^*^** | 3.28 (1.07–10.17) | 0.038**^**^** |
| BA PI, quartile^b)^ | | | | |
| Q1 (0.42–0.80) | 1 |  | 1 |  |
| Q2 (0.80–0.94) | 0.97 (0.50–1.89) | 0.935 | 0.93 (0.45–1.90) | 0.841 |
| Q3 (0.94–1.10) | 1.23 (0.66–2.28) | 0.518 | 1.05 (0.52–2.15) | 0.890 |
| Q4 (1.10–2.50) | 2.22 (1.16–4.26) | 0.016^**^ | 2.39 (1.10–5.25) | 0.028**^**^** |

BA PI measured by transcranial Doppler ultrasonography.

OR, odds ratio; CI, confidence interval; BA PI, basilar artery pulsatility index.

^a)^Adjusted by age, sex, history of atrial fibrillation, current smoking status, National Institutes of Health Stroke Scale at admission, systolic blood pressure, serum homocysteine level, cerebral atherosclerosis score, and white matter hyperintensity lesion. ^b)^Group was divided into quartiles based on BA PI.

^*^P < 0.01, **^**^**P < 0.05.
